# Avoiding the pitfalls of adaptive management implementation in Swedish silviculture

**DOI:** 10.1007/s13280-015-0750-9

**Published:** 2016-01-07

**Authors:** Lucy Rist, Adam Felton, Erland Mårald, Lars Samuelsson, Tomas Lundmark, Ola Rosvall

**Affiliations:** Swedish University of Agricultural Sciences, 90183 Umeå, Sweden; Southern Swedish Forest Research Centre, Box 49, 23053 Alnarp, Sweden; Umeå University, 901 87 Umeå, Sweden; Rosvall Forest Consulting AB, Ringvägen 43, 91832 Sävar, Sweden

**Keywords:** Climate change, Forest management, Risk, Silviculture, Uncertainty

## Abstract

There is a growing demand for alternatives to Sweden’s current dominant silvicultural system, driven by a desire to raise biomass production, meet environmental goals and mitigate climate change. However, moving towards diversified forest management that deviates from well established silvicultural practices carries many uncertainties and risks. Adaptive management is often suggested as an effective means of managing in the context of such complexities. Yet there has been scepticism over its appropriateness in cases characterised by large spatial extents, extended temporal scales and complex land ownership—characteristics typical of Swedish forestry. Drawing on published research, including a new paradigm for adaptive management, we indicate how common pitfalls can be avoided during implementation. We indicate the investment, infrastructure, and considerations necessary to benefit from adaptive management. In doing so, we show how this approach could offer a pragmatic operational model for managing the uncertainties, risks and obstacles associated with new silvicultural systems and the challenges facing Swedish forestry.

## Introduction

Since the 1950s Swedish forests have primarily been managed using rotational clear-cutting of even-aged conifer-dominated stands (SFA 2009). In recent years, increasing pressure for more diversified management has been coming from multiple directions, including governance trends related to climate change, energy supply and environmental protection (Sandström and Sténs 2015). There are also vulnerabilities and uncertainties related to climate change that will require enhanced adaptive capacity (Larsson et al. 2009, 2014). The adoption of a more diversified silviculture, including changes to the methods by which forests are managed, harvested and regenerated, will likely be needed to meet these challenges and the increasing variety of demands placed on them by society.

However, applying new methods also carries significant uncertainties and risks, for example, those associated with the projected performance of management (Seidl and Lexer 2013) or potential adverse impacts on forest values other than timber or biomass (Gamfeldt et al. 2013). Forest owners or managers may be hindered in adopting new methods by ecological complexity and uncertainty, as well as uncertainties regarding changing trends and markets for forest products (Ulmanen et al. 2012). Inflexible legislation (due to the requirement that management must be based on scientific evidence or proven experience) and tradition, or culture among professional silviculturalists may also be obstacles (Larsson et al. 2009; Puettmann et al. 2015; SFA 2015).

Expansion of the range of silvicultural methods requires a rigorous means of evaluation including consideration of risk and uncertainty in long-term planning (Yousefpour et al. 2011). Adaptive management (AM) (Holling 1978; Walters 1986) is advocated as a means to tackle some of these challenges (Yousefpour et al. 2011; Temperli et al. 2012; Felton et al. 2013) and is now being actively considered by the Swedish government (SFA 2013b; Larson et al. 2014). In November 2012, the Swedish Forest Agency (SFA) and the Swedish University of Agricultural Sciences (SLU) were asked by the Swedish government to evaluate the potential for implementing Adaptive Forest Management (AFM) in Sweden, opening a window for greater experimentation. The expectation is that this model of AM will allow assessing new silvicultural methods that might better meet the joint policy goals of biomass production and environmental status, for which, however, there is currently limited practical experience or scientific knowledge (SFA 2013b). Given that the benefits of AM are desired, and its implementation in Swedish forestry is being actively planned for, there is a need to reflect and learn from documented previous experience with AM, as well as the latest advances in the AM paradigm to help ensure the best possible outcomes (Rist et al. 2013).

The current Swedish government initiative differs from many previous experiences with AM in that such a program is being envisaged to take place at a national level with many forest owners involved. Previous large-scale implementation of AM has taken place mainly on government-owned land, for example, The Northwest Forest Plan (NWFP) in the Pacific Northwest region of the United States. The NWFP aimed initially to protect critical habitat for the northern spotted owl (*Strix occidentalis caurina*) and later came to deal with sustainable forestry, non-timber forest products and habitat protection more generally. The plan is still in operation today 20 years later (Bormann et al. 2007; Winkel 2014; Davis et al. 2015). In Sweden, use of AM to date has also been at smaller scales with a focus on co-management processes rather than technical learning and experimentation (Sandström pers. comm.).

Thus, specific guidance is therefore needed to highlight and help resolve the challenges of applying AM in a context characterised by large spatial and temporal scales, and multiple land tenures. In this paper, we offer such guidance by reviewing the major pitfalls likely to be experienced during vital stages of AM implementation, and provide a potential means of navigating these hazards. Our focus is specifically on implementation of an AM process (technical learning), rather than the policy and governance process within which management is embedded (institutional learning). Furthermore, whereas we cannot provide prescriptive answers regarding issues to be determined by the AM process itself and its participating forest owners and stakeholders, we can provide guidance by synthesising up to date and relevant knowledge regarding common pitfalls and potential solutions relevant to successful AM implementation and goal fulfilment. We indicate the investment and infrastructure necessary to benefit from AM and highlight key issues requiring particular attention and long-term sustained commitment. In doing so, we show how AM could indeed provide a model for managing the uncertainties, risks and obstacles associated with the establishment and use of new silvicultural methods in Swedish production forestry.

## Swedish forestry

The dominant silvicultural system in Sweden is one of clear-cutting with a rotation length varying from 50 to 140 years. This system aims to maintain an even age-class distribution and a steady flow of timber, while taking into account other ecosystem services at the stand and landscape levels. Timber production is primarily achieved using two native conifer species, Norway spruce (*Picea abies*) and Scots pine (*Pinus sylvestris*). Approximately half of all of Sweden’s forests are owned by private individuals but ownership structure varies significantly between different parts of the country. In the north, the proportion of forests owned by the state and private corporations is much higher than in the south. Forest owners and government agencies are interested in a range of alternative silvicultural methods and systems including, for example, the use of introduced or improved tree species, broadleaf mixtures, shortened rotation times, increasing fertilisation intensity, continuous cover forestry, short-rotation bioenergy, and forest residue extraction (Sandström et al. 2011; Dahl et al. 2014).

The Swedish Forestry Act requires equal consideration of timber production and environmental values in managed forests, and approximately 75 % of the Swedish forestland is actively managed with this multiple-goal perspective (SFA 2013a). Paralleling the development of Swedish forest policy, there has also been a rapid increase in forest certification within Sweden, resulting in over 70 % of the managed forest certified according FSC (Forest Stewardship Council) and/or PEFC (Program for the Endorsement of Forest Certification). While stated to be of equal importance in Swedish law, in practice production is often prioritised over biodiversity (Ulmanen et al. 2012; SFA 2013a); for example, many intensive methods to increase forest productivity have been found to have adverse implications for forest biodiversity (e.g. Ranius and Roberge 2011). The 1993 Forestry Act is based on the principle of “Freedom-under-responsibility” and is more oriented towards overall goals than specific regulations (Kjellin 2001). In practice, this means that forest owners have considerable autonomy in how to manage their forest as long as economic viability and biodiversity are both taken into account and that the applied methods are well known and based on scientific evidence or proven experience. In terms of diversification options, the industry currently focuses on exploring changes in silvicultural methods (e.g. biomass extraction, fertilisation or shortened rotation times), while placing less focus on alternatives that may be more consistent with multi-use goals, i.e. alternatives to a clear-cutting system (Gustafsson et al. 2012; Felton et al. 2016).

The capacity to deliver multiple ecosystem services is a need highlighted in forest policy discussions (e.g. Larson et al. 2014) yet there is little specific reference to many social and aesthetic values. In addition, a narrow range of actors (i.e. those owners who decide to engage in the application of new methods) still seem to characterise decision-making processes (Sandström and Sténs 2015). Thus, new approaches are recognised as needed to tackle the challenges associated with managing for multiple goals, as well as navigating the ecological uncertainties of management and new pressures from climatic change related trends.

## Adaptive management

Adaptive management (AM) (Holling 1978; Walters 1986) has been put forward as a way of managing natural resources in the face of uncertainty. Emerging originally as an experimental and rather quantitative approach to reduce uncertainty in management, AM’s appeal has led to a broadening of interpretations to include the social, political, and institutional contexts of management, for example, with ideas of adaptive co-management and adaptive governance. Here, we focus specifically on the original concept with its focus on experimental management and technical learning.

AM is management that purposely and explicitly reduces ecological uncertainty (Holling 1978; Walters 1986). It embraces the idea that where knowledge about potential management choices is lacking, the best way to learn about those choices is through direct comparisons of their performance in the field, i.e. through planned experimentation (Walters 2007). In addition, AM emphasises collaboration and participation, both to reduce potential conflicts and enhance the base of knowledge contributing to the process (Holling 1978).

Since the emergence of AM it has been discussed and applied by varying groups including scientists, politicians, resource management authorities, etc., all motivated by different objectives and thus bringing different points of focus. Significant disagreements and confusion exist in the literature regarding the suitable circumstances for AM application (Rist et al. 2012). For example, the presence of highly controversial risks, extended temporal or spatial scales, or high uncertainty regarding the biological and ecological relationships that drive resource dynamics, have all been judged to predispose to the likelihood of an unsuccessful application of AM (e.g. Gregory et al. 2006; Doremus et al. 2011). As a result, some suggest that AM is not well suited to tackle the type of large complex uncertainties that the shift of an entire production forestry system may entail (Norgaard et al. 2009). However, Rist et al. (2013) showed that much of the pessimism surrounding AM lacked empirical support and presented a new framework for deciding when AM is appropriate, feasible, and subsequently successful. This framework shows that there are no categorical limitations to AM’s appropriate use, the boundaries of application being defined by problem conception and the resources available to managers. Rist et al. (2013) highlighted AM as a technical management tool, thus separating the concept from the burden of failures that result from the complex policy, social, and institutional environment within which management overall occurs. This new paradigm suggests that AM can handle complex and complicated environmental problems, given adequate resources and a suitable breakdown of the targeted uncertainties (Rist et al. 2013). Thus, the challenge is to provide adequate guidance for AM implementation, drawing from this enhanced theoretical understanding as well as lessons learnt to date from its implementation (e.g. Lee 1999; Shea et al. 2002; Reeves et al. 2006; Bormann et al. 2007; Davis et al. 2015).

## Avoiding common pitfalls

**Fig. 1:**
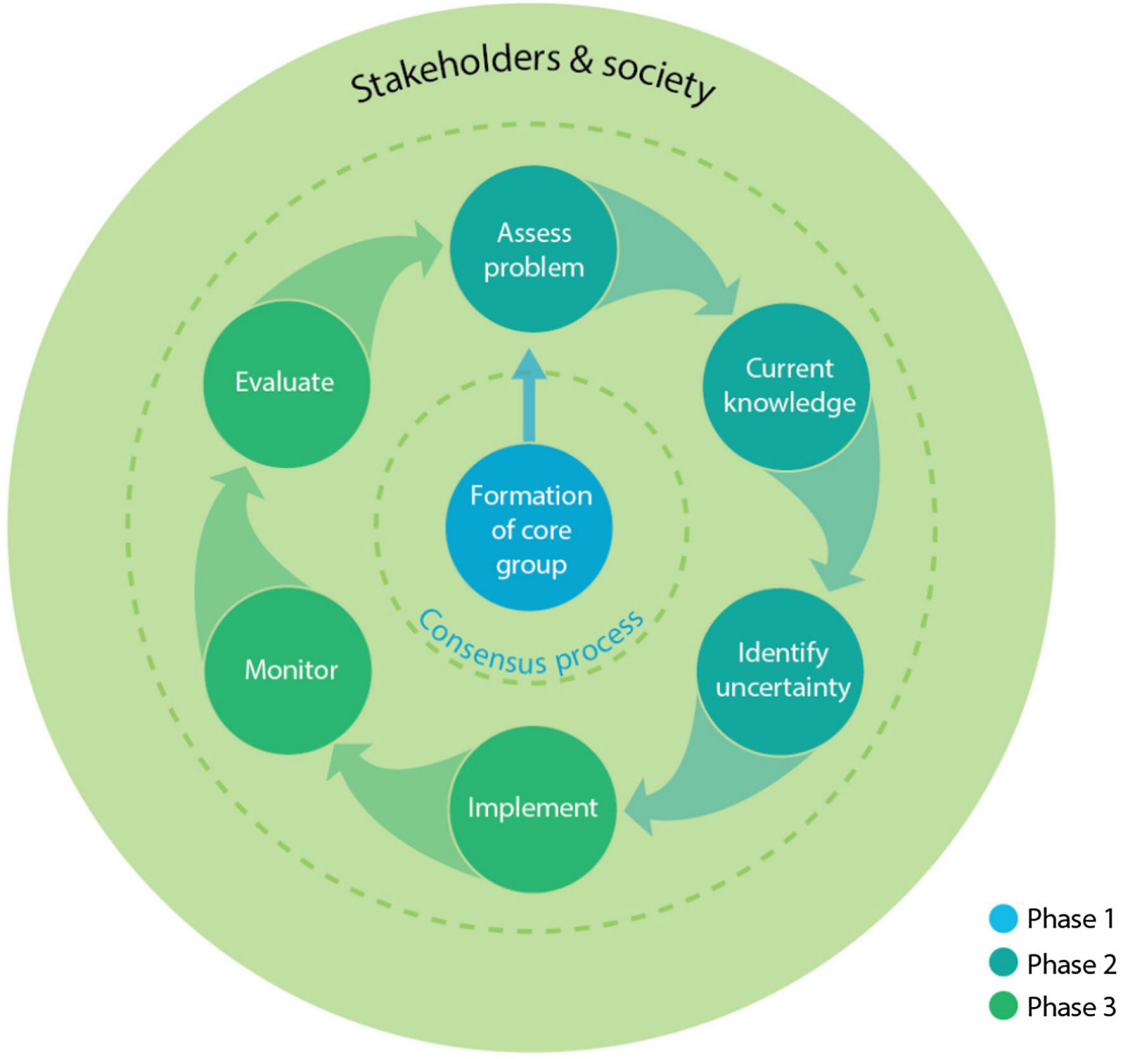
The AM process including initiation, planning and operationalisation

We assume that while a central institution (“core group”) would provide support and coordination for implementation efforts, practical implementation of new silvicultural methods would ultimately be the choice and responsibility of those forest owners (including the state, private individuals and forest companies) who decide to engage in such a new opportunity—we refer to these as “owners”. Others engaged in, but not necessarily directly involved in implementation, for example, nature conservation groups—are referred to as “stakeholders”. A central institution would offer a “container” for the AM process and a meeting place for authorities, owners and stakeholders. Below we set out key issues, guidelines and tasks according to three phases: (1) initiation; (2) planning and preparation; and finally, (3) operationalisation (Fig. 1). It should also be noted that the boundaries between phases are overlapping and therefore should not be viewed too rigidly.

### Phase 1: Initiation of adaptive management

#### Acknowledge constraints and the need to coordinate efforts

Coordinated, landscape-scale action will be necessary for any significant AM effort in forestry, which to be effective will need to occur across areas under different tenures and managed by different organisations or private individuals with different priorities and values. Approximately 50 % of forestland is privately owned in Sweden, a major contrast to examples of AM implementation in, for example, the US and Canada, across large areas of public land (e.g. Moir and Block 2001; Davis et al. 2015). Multiple private landowners (or their representative organisations) would therefore be central participants in the AM process.

The participation of other stakeholders, particularly those who may lack trust in the nature of the process, may be more of a challenge. For example, where there is a history of distrust between environmental organisations and governmental bodies, environmental organisations may decline involvement altogether. Similarly, industry may not see it as being in their interest to participate in a process likely requiring compromise between environmental and production interests. Such an absence may greatly decrease the likelihood that the core group is, or is perceived to be, an organisation representative of relevant actors’ interests. There is no simple answer to such challenges (Bodin et al. 2006; Reed 2008; Stankey and Henry 2009). Building trust is unlikely to be achieved if there are inherent power imbalances in the process itself, for instance in defining what are or are not considered to be unacceptable outcomes (phase 2). This is particularly relevant in the context of more intensive forestry measures if ecological risks are not adequately accounted for in experimental management plans or monitoring and evaluation protocols. Trust is especially likely to be lost if the AM process is suspected to be merely a conciliatory paradigm employed to ease the transition towards controversial forestry practices, such as in the advocated expansion of the use of intensive fertilisation or introduced tree species. Participation must therefore take place from the earliest stages and in all subsequent planning, monitoring and evaluation (McLain and Lee 1996; Ludwig et al. 2001; Stringer et al. 2006).

Power imbalances may likewise create disincentives for other stakeholders to participate. Participation is unlikely if the current status quo is considered more desirable than being seen as participating in an AM process that results in decisions which conflict with their interests or established positions. Unfortunately there is an inherent potential for alienating some stakeholder groups. Those forest owners and stakeholders who are generally empowered may avoid processes that try to redistribute power, whereas those who are disempowered may see little benefit in joining a process in which such imbalances are not rectified. Such efforts are nevertheless vital if the AM process is to be as effective as possible.

AM was originally conceived as a process dependent on a small, predominantly scientific core group with cross-disciplinary expertise acting as a node for integrating information and coordinating management action and evaluation processes (Holling 1978). For the reasons described above, we envisage that in order to increase the likelihood that AM is successfully implemented, the core group may need to be created as a new independent institution. In will also need to embrace a more heterogeneous group of actors with diverse interests (Plummer and Armitage 2010). The process by which group members are selected and according to which criteria also needs careful thought to ensure involvement and interaction with the full range of national actors. This is similarly the case in processes of decision-making and communication. In a review of AM case studies from North America and Europe, Gunderson et al. (1995) reported the “extreme nature of the recalcitrance or inertia of institutions, and the almost pathological inability to renew or restructure”. Were the core group to be established within an existing organisation, the risk of such ‘recalcitrance’ may be higher.

Key to success in the shorter term would be that the new institution has legitimacy, independence and is free of a limiting institutional history. In the longer term, institutional flexibility and the ability to transform and adapt will be key (Young 2009). This does not rule out the possibility that successful outcomes can be achieved by AM units created within existing organisations, merely that legitimate concerns can be raised regarding their potential for success, especially if the processes that have led to past failures are not actively acknowledged and dealt with. Either way, the group leading implementation must be methodical in their approach to its design and implementation. This includes committing to substantive management criteria, engaging fully in actor participation, and resisting the temptation to employ AM to dodge burdensome forestry regulatory requirements.

#### Ensure a clear understanding of AM

With owners voluntarily joining the AM process, its success requires that participants recognise AM as an appropriate and potentially effective means of addressing the management objective to explore potential alternative silvicultural methods. For this to take place, sufficient understanding of what is entailed in an AM process is required by these individuals and groups. Such understanding helps to ensure that unexpected and potentially undesirable experimental outcomes will be seen as a source of learning and to improve subsequent decision-making, rather than as liabilities to the AM process (Allen and Gunderson 2011). History shows that where this does not occur, and those involved are not adequately familiar with the purpose of AM, or otherwise not committed to a learning process, effective learning and decision-making will be limited (Walters 1997; Bormann et al. 2007).

An explicit but simple formulation of the task facing the core group, and those owners involved as active participants, may be required to assist with this. For example, the task might be articulated as “to explore a range of silvicultural alternatives and assess the degrees to which they perform according to specified criteria, including, for example, in relation to production and impacts on alternative forest values”. By grounding the task in simple terms a common focus to gather around can be provided.

##### Policy obstacles

AM implementation may also face obstacles in terms of existing regulatory frameworks (Benson and Stone 2013). The current legal framework in Sweden is considered by some to lack the flexibility that is required to develop, discuss and implement alternative silvicultural systems or new measures (Larsson et al. 2009). This static nature may actually enhance the vulnerability of forests (e.g. to climate change) or weaken opportunities to respond to new demands. But reforms would require a careful balance to retain important restraints on the use of methods that could be detrimental to other forest values, while still permitting space for those alternatives that are chosen for experimentation. Furthermore, the current political context may not provide the incentives necessary to engage forest owners in an AM program that requires the cooperation of a multitude of heterogeneous actors (Westley et al. 2010). To some extent, reform of the current legal framework, or how the framework is applied, may be required in order to facilitate AM (Ruhl 2008; Ruhl and Fischman 2010; Benson and Stone 2013), in particular in relation to effective monitoring (SFA 2013b), and overall support for broader participation. In addition, that AM procedures cannot substitute for showing that a plan will meet substantive management criteria required by law must be recognised (Ruhl and Fischman 2010).

### Phase 2: Planning and preparation

#### Identify key ecological uncertainties

The assimilation of existing knowledge is a core element of AM. This would involve identifying relevant individual, institutional, and published sources of knowledge regarding potential alternatives, including the bringing together of representatives of organisations that are potential sources of such knowledge. Discussion to clarify where understanding is solid and where it is more limited would be required, for example, on specific silvicultural methods in terms of production values or potential ecological impacts. Such a process of knowledge gathering illuminates more clearly the priority uncertainties and would include elaboration of potential management actions, indicators, as well as time horizons and spatial extents (Holling 1978).

For example, in relation to the use of introduced tree species as one potential new silvicultural alternative with a focus towards climate adaptation, many candidate species are already planted to greater or lesser extents in different parts of the country (Forest Europe 2011). Some stands are close to maturity and potentially able, with the appropriate study design, to provide relevant information. Information on issues including invasive risk in surrounding habitats, pest or pathogen occurrence, hybridisation potential, susceptibility to browsing pressure, as well as growth rates and production values (Felton et al. 2013). Information relevant to filling knowledge gaps is likely to be dispersed among private forest owners, forest companies, other actor groups and the scientific literature as well as senior researchers at government institutions and universities, emphasising the key coordination role of the core group in bringing available knowledge together.

The identification of those uncertainties most important to management is a key stage. Conceptual models may be used to indicate biological or physical variables, processes or parameters as well as management interventions that are sources of uncertainty with regard to specific silvicultural alternatives (Holling 1978; Walters 1986). Such models can then form the basis for on-going learning (Holling 1978; Walters 1986). For example, in the context of increasing fertilisation intensity, a simple model may represent associations among fertilisation intensity, annual growth, forest structure and leakage of nitrogen to groundwater. Contrasting hypotheses about the impact of fertilisation on annual growth are then easily incorporated into different versions of the model by describing different functional relations between fertilisation intensity and tree growth rates. In addition, contrasting hypotheses about the influence of fertilisation on biodiversity values can be incorporated by describing species diversity and richness in terms of either fertilisation directly, or forest structure indirectly. In combination, these hypotheses define different models, each with its own predictions about fertilisation impacts and each with its own measure of confidence that evolves over time. The models and their measures of confidence characterise structural uncertainty, which is reduced as management actions are taken and monitoring data are used to update the confidence measures. Learning is expressed through the updating of these measures and can be taken forward in, for example, an annual process of setting fertilisation protocol advice or guidelines.

#### Monitoring

Monitoring programs are crucial to effective AM (Holling 1978; Bormann et al. 2007). A central tenet of the AM paradigm is that monitoring has to be adequate to detect change resulting from the management applied. It therefore follows that where management effects accrue over long time periods, coordinated monitoring will also have to occur over a long period of time (Lindenmayer and Likens 2010a). Replicated experimental designs, no matter how elegant and rigorously implemented, may substantially fail the test of management relevance, if the integrated effects of dynamics that vary idiosyncratically over multiple time and spatial scales are not experienced (Russell-Smith et al. 2003). Continuing with the fertilisation example above, testing the predictions of competing models that aim to assess the potential for negative biodiversity impacts, may require repeated frequent monitoring over the space of a few years. Other methods or interventions may require monitoring over a number of decades. To achieve this, in addition to explicit experimental designs, AM requires the specification and documentation of comprehensive monitoring protocols, including individual owners and actors roles, relationships, and responsibilities in the implementation, assessment and evaluation of management. A key role of the core group will be to meet these needs, including, for example, in the development of Standard Operating Procedures and producing specific monitoring protocols to facilitate standardised implementation as well as collection and collation of data and coordination among the organisations or individuals involved.

Without securing a long-term commitment to provide adequate financial support, the processes knowledge-basis, AM cannot succeed. This investment and commitment to monitoring will have to be adequately scaled to the nature of the uncertainty being addressed (Bormann et al. 2007). Due to the repeated calls for investment in environmental monitoring programmes over previous decades, it may be possible for AM monitoring programs to make some use of established monitoring protocols, expertise and infrastructure, rather than developing parallel systems. The recently established Swedish Infrastructure for Ecosystem Science (SITES) could have a role to play, as could building structured and replicated experiments around ongoing forest management operations (e.g. Bunnell and Dunsworth 2009; Lindenmayer and Likens 2010b).

For Swedish forestry, the spatial scale is extensive, the temporal scale protracted, and the information sources scattered among various forest owners, forest organisations and other actors. A failure to adequately account for such logistical obstacles, and specifically to coordinate monitoring methodologies, will make it unlikely that AM can meet expectations. There is an extensive literature on how to develop effective monitoring programs that would require detailed consultation if the many associated obstacles and pitfalls are to be avoided (e.g. Lindenmayer and Likens 2010a, b). As well as status and trend monitoring with a focus on changing physical, chemical, or biological attributes over time, implementation and effectiveness monitoring will also be needed. Implementation monitoring to ensure that the work that was proposed was actually completed successfully. Effectiveness monitoring to evaluate whether the overall objectives have been met, including at different scales. For example, a full monitoring system might include a reporting template for monitoring status or trends as well as a report card that owners use to self-report information related to compliance with the requirements of the monitoring program. A third-party audit system that assesses the reliability of owners’ self-reporting may also be used.

#### Response variables and decision rules

Selecting specific variables for an assessment and monitoring framework requires the incorporation of the values and risks of relevant parties, while recognising that perceptions of risk will most likely vary across stakeholder groups (Weiss 2001). Variable choice is thus a value-laden process, and consequently, the selection of variables usually reflects the values of the main participants. This further highlights the importance of representation and participation mechanisms (phase 1). Management actions and experiments must also be designed and planned such that they relate directly to reducing the uncertainties they are intended to address (Lindenmayer and Likens 2010a). For example, experiments must match operational approaches and scales in order to permit direct transfer of these efforts to the actions of forest managers (Walters and Holling 1990).

AM also requires ‘decision rules’ or stopping thresholds and a priori agreement on target values (Holling 1978; Walters 1986). When changes are detected, for example, those relating to potentially negative impacts on ecological forest values, specific decision rules must already be in place, which determine what actions are to be taken at what point. These decision rules need to be in relation to the uncertainties and areas of knowledge that have been prioritised. For example, in trying to tackle the challenge of climate change, managers need to be actively aware of the risk that efforts could exacerbate other environmental problems, such as biodiversity loss—thereby creating the so-called “bio-perversities” (Lindenmayer et al. 2012). Such trade-offs could arise in Sweden if efforts to adapt to or mitigate climate change, come into conflict with the substantial efforts that have been directed towards improving habitat suitability in production forests. For example, logging residue extraction for bioenergy production can readily come into conflict with efforts to increase dead-wood availability in production stands (Ranius et al. 2014).

Likewise, the increased use of introduced tree species could result in the invasion of sensitive ecosystems, exacerbate pest and pathogen outbreaks, or directly challenge the effectiveness of conservation considerations (e.g. green tree retention) taking place in production forests (Felton et al. 2013). Thus, if we decide to implement a new silvicultural measure with the objective to learn more about its production values and impacts on biodiversity, then we must decide in advance what form and level of undesirable outcome is sufficient to cease or greatly limit the further use of that method. Thresholds may also be determined in relation to disappointing growth rates and wood-quality values which if crossed, would also require a corresponding shift in management strategies. Such decision rules would require significant discussions via the core group and wider consultation processes with stakeholders given the inherent choices regarding what and whose values are prioritised and what choices are made regarding potential risks and benefits (Leach et al. 2010).

An additional key challenge related to institutional learning rather than technical learning, is whether the decision-making structure of adaptive management can itself be adaptive; that is, whether the knowledge and experience gained in its application can be reflected in higher-level structural adjustments where needed, including those gained with the inputs from experts and the public. Unravelling these policy and institutional issues will require innovative mechanisms for producing effective dialogue and new ways of handling disputes within a process that all parties regard as fair (Mårald et al. 2015).

### Phase 3: Operationalisation of adaptive management

#### Knowledge transfer

While supported by the core group and other organisations, practical implementation of new methods will ultimately be the responsibility of forest owners including the state, private individuals and forest companies. Thus, clear documentation including standard operating procedures and effective channels of communication will be required to maximise the potential for feedback and learning in practice. Some operational tasks may take place as management directly implemented by owners, others as supporting research or coordination managed by the core group. Several organisations may already be well positioned to tackle some of the prioritised uncertainties, including those based on active experimentation, natural experiments, monitoring, modelling, and simulations. Others may have the communication experience and networks to contribute with.

The literature is rather clear on the important role of institutions in implementing AM and the need for new approaches are needed in this respect (Gunderson 1999). However, it pays less attention to the question of precisely what types of institutional structures and processes are required for success, particularly in relation to large spatial contexts, extended temporal durations and complex land ownership. McLain and Lee (1996) argue that the rationale for adaptive learning in management systems rests on three key elements: (1) rapid knowledge acquisition; (2) effective information flow; and (3) processes for creating shared understandings. Structures and processes that encompass these elements would be needed, including mechanisms for communication between the core group and implementing forest owners. Specifically, these would need to deal with integration, data availability and the time intervals of management decision-making. There may in fact be a need to explore alternative institutional structures and processes, such as how to integrate local knowledge and alternative values into decision-making processes, in order to identify those processes most likely to be successful. The core group should thus be nested across scales and governance levels (Plummer and Armitage 2010). Attention to the growing adaptive co-management and environmental governance literature, including in relation to institutional learning, could provide valuable further guidance (e.g. Armitage et al. 2009; Plummer 2013).

**Fig. 2:**
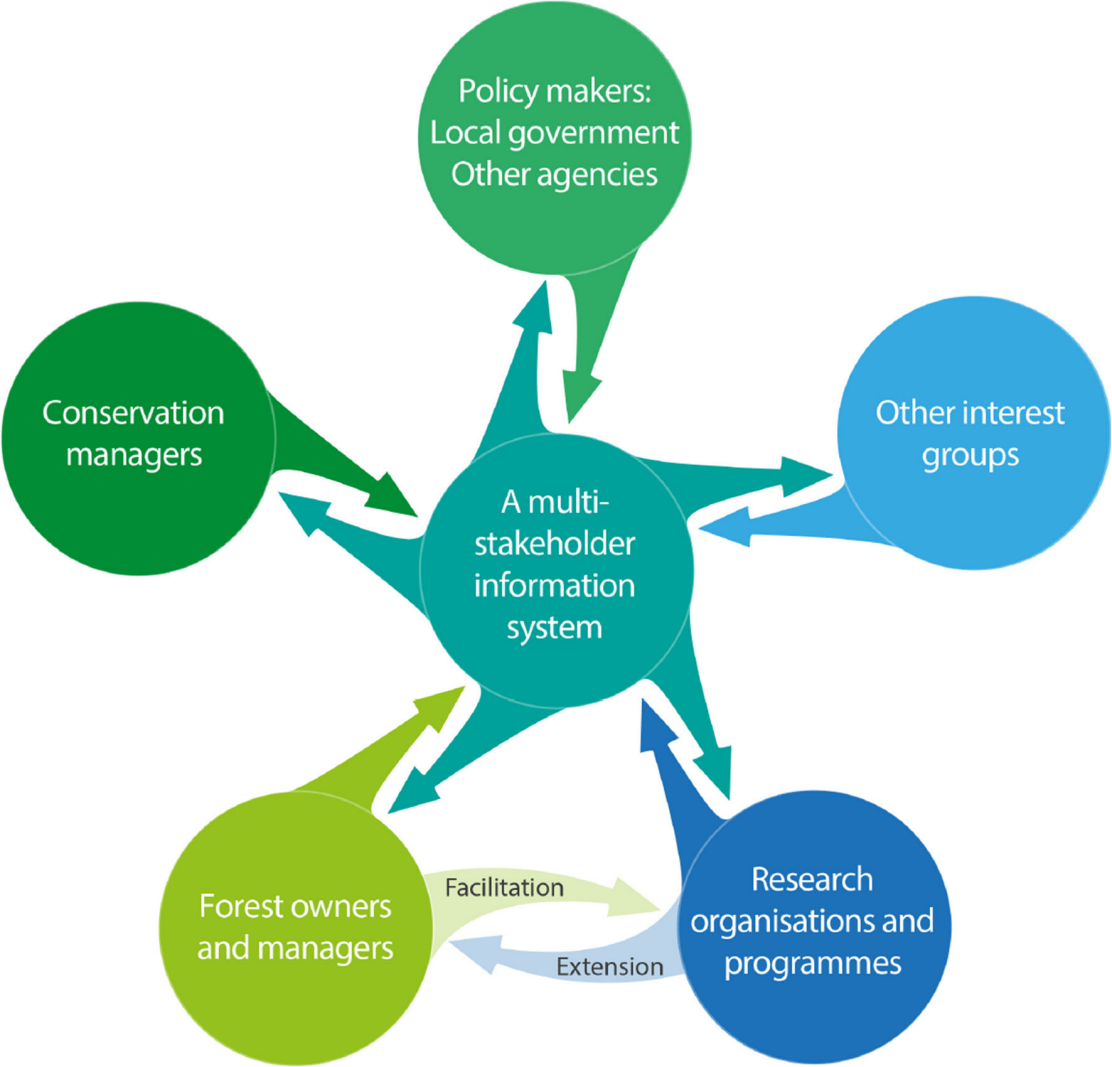
A Multi-Stakeholder Information System offering a network for information providers and users. Note that while all actor groups are involved, roles will vary, and some groups may feature more prominently and contribute more substantially to the process than others

Research on knowledge systems for meeting sustainability objectives has shown that managing boundaries between knowledge and action in ways that simultaneously enhance the salience, credibility and legitimacy of the information they produce are key (Cash et al. 2003). To be effective, the institutional mechanisms in this process will need to facilitate active, iterative, and inclusive communication with owners and other stakeholders; open channels of communication and shared understandings, as well as active mediation of conflicts (Cash et al. 2003). In practical terms, mechanisms of coordination including specific implementation guidelines and protocols for management (see phase 2) will be needed. For example, establishment of a ‘Multi-Stakeholder Information System’ to provide background ecological knowledge and best practice guidelines, as well as other functions, may be advisable (Fig. 2). Such an information system could draw on the experience and knowledge of all relevant actors. It could also be Internet-based (Allen et al. 2001), and could be designed as an open-ended platform that can be continually updated as new information becomes available through research and monitoring. In this way, different groups or organisations could maintain control over their own information, while sharing a common ‘gateway’ (Fig. 2).

#### Scale of adoption

Risk spreading may be a desirable way of addressing uncertainty in relation to climate change associated threats to forestry (Lindner et al. 2014). In such a case, identifying target levels of diversification (of silvicultural measures) and which owners contribute to those targets would be needed. Without effective management options for reducing vulnerabilities, and suitable scales of implementation, the true extent of the challenge will be left unidentified, making it highly likely that responses will be disproportionate, insufficient, or simply misguided.

Funding is likely to be a major issue throughout all three phases. Long-term government support earmarked for, for example, climate change adaptation objectives or the potential new National Forest Program would facilitate the work of the core group itself and the process more broadly (SOU 2007; SFA 2013c), yet current indications suggest this is unlikely. Some forest owners may be willing to participate, perhaps motivated by the potential to increase forestry profits, but government financing will be required to support owners in monitoring and evaluation costs, particularly where actions are motivated by values other than production, e.g. biodiversity conservation (Bormann 2007). A discussion also relevant to core funding regards the responsibility for unforeseen or undesired impacts. For example, if private industry wishes to try an introduced species and that species later establishes substantial populations within protected areas, who is then responsible for eradication efforts if they are deemed necessary?

#### Societal acceptance

Additional challenges relate to how the technical process of AM can be embedded within a broader framework that deals with the social, political, and institutional context of management. While we focus on technical learning it is also important to recognise the intersection between technical (management) and institutional (governance) learning. The needs for wider involvement is also supported empirically by earlier experience with AM implementation (e.g. Walters 2007), including within Sweden (Danell and Bergström 2010). Further support still coming from the move towards more post normal ideas of science whereby the research process becomes open to involvement by others. In this way, science is used in a way in which it is “no longer imagined as delivering truth” and instead, decision-making becomes a mutual learning process among different stakeholders (Funtowicz and Ravetz 1993). The intended purpose of AM is not to enable the negotiation of political priorities or resolution of conflict, it nevertheless remains a sociopolitical action as well as a scientific and technical exercise in that different actors and perspectives are involved (Stankey et al. 2005).

## Conclusions

While operationalisation of AM may appear to be a significant undertaking, the alternative for Swedish forestry is to continue with a piecemeal approach to the use of alternative silvicultural measures including those focused on climate change adaptation or enhanced biodiversity status. The former introduction of lodgepole pine (*Pinus contorta*) in Swedish forestry can be seen as such an example—with initially a narrow focus and little coordinated learning (Engelmark et al. 2001). Such a piecemeal or incremental approach can in fact represent a further trap where the inherent nonlinearities, thresholds, time delays and spatial distributions of ecological systems may hide the potential effects that would result from a larger intervention (Holling 1978). Thus, while there are certainly major challenges and costs associated with attempting to implement the AM process, these costs may well be exceeded by the consequences of inaction. Forest owners and managers need to know where alternatives to the current system of clear-cutting or alternative silvicultural methods are feasible, economical and ecologically sound, and learning in this respect can best be achieved via joint and coordinated efforts.

The AM process may be resource intensive, but the alternative piecemeal approach to natural resource management often results in undesirable outcomes. A half-hearted approach to AM could, however, result in a worst-of-both-worlds outcome. This can occur when governments or agencies desire the benefits of AM without fully committing the resources necessary for its correct implementation. Then the AM label may be affixed to management procedures only superficially related to AM, which may result in (i) substantial costs in terms of time and resources; (ii) negligible benefit, or even detrimental to natural resources and the ecosystem services they provide; and (iii) loss of public confidence and support for future legitimate attempts at instigating real AM.

We have highlighted key issues, guidelines and tasks in an AM process requiring adequate foresight and long-term sustained commitment; drawing on the AM literature to highlight important “lessons learnt” and suggesting how likely pitfalls can be avoided. We show how AM could indeed provide a pragmatic operational model for managing the uncertainties associated with the establishment and use of new silvicultural systems and methods in Swedish production forestry. In addition, appropriately resourced, the implementation of AM in Swedish forestry offers an exciting opportunity to simultaneously evaluate how this approach to deal with uncertainty can be successfully applied to large spatial extents, management for extended time-scales in the context of complex land tenures.

## References

[CR1] Allen WJ, Bosch OJH, Kilvington MJ, Oliver J (2001). Benefits of collaborative learning for environmental management: Applying the integrated systems for knowledge management approach to support animal pest control. Environmental Management.

[CR2] Allen CR, Gunderson LH (2011). Pathology and failure in the design and implementation of adaptive management. Journal of Environmental Management.

[CR3] Armitage DR, Plummer R, Berkes F, Arthur RI, Charles AT, Davidson-Hunt IJ, Diduck AP, Doubleday NC (2009). Adaptive co-management for social–ecological complexity. Frontiers Ecology and Environment.

[CR4] Benson MH, Stone AB (2013). Practitioner perceptions of adaptive management implementation in the United States. Ecology and Society.

[CR5] Bodin Ö, Crona B, Ernstson H (2006). Social networks in natural resource management: What is there to learn from a structural perspective?. Ecology and Society.

[CR6] Bormann BT, Haynes RW, Martin JR (2007). Adaptive management of forest ecosystems: Did some rubber hit the road?. BioScience.

[CR7] Bunnell FL, Dunsworth GB (2009). Forestry and biodiversity. Learning how to sustain biodiversity in managed forests.

[CR8] Cash DW, Clark WC, Alcock F, Dickson NM, Eckley N, Guston DH, Jäger J, Mitchell RB (2003). Knowledge systems for sustainable development. Proceedings of the National Academy of Science of the United States of America.

[CR9] Dahl E, Lobo A, Myking T (2014). The role of exotic tree species in Nordic forestry. Scandinavian Journal of Forest Research.

[CR10] Danell K, Bergström R (2010). Wildlife, man and society.

[CR11] Davis, J., J.L. Ohmann, R.E. Kennedy, W.B. Cohen, M.J. Gregory, Z. Yang, H.M. Roberts, A.N. Gray, and T.A. Spies. 2015. *Northwest Forest Plan*–*the first 20* *years (1994*–*2013): Status and trends of late*-*successional and old*-*growth forests.* General Technical Report Portland, OR: U.S. Department of Agriculture, Forest Service, Pacific Northwest Research Station.

[CR12] Doremus, H., W.L. Andreen, A. Camacho, D.A. Faber, R.L. Glicksam, D.D. Goble, B.C. Karkkainen, D. Rohlf, et al. 2011. *Making good use of adaptive management*. Center for Progressive Reform White Paper No. 1104.

[CR13] Engelmark O, Sjöberg K, Andersson B, Rosvall O, Ågren GI, Baker WL, Barklund P, Björkman C (2001). Ecological effects and management aspects of an exotic tree species: The case of lodgepole pine in Sweden. Forest Ecology and Management.

[CR14] Felton A, Boberg J, Björkman C, Widenfalk O (2013). Identifying and managing the ecological risks of using introduced tree species in Sweden’s production forestry. Forest Ecology and Management.

[CR15] Felton A, Gustafsson L, Roberge J-M, Ranius T, Hjältén J, Rudolphi J, Lindbladh M, Weslien J (2016). How climate change adaptation and mitigation strategies can threaten or enhance the biodiversity of production forests: Insights from Sweden. Biological Conservation.

[CR17] Forest Europe. 2011. *State of Europe’s Forests 2011: Status and Trends in Sustainable Forest Management in Europe*. Ministerial Conference on the Protection of Forests in Europe, Oslo, p. 344.

[CR19] Funtowicz SO, Ravetz JR (1993). Science for the post-normal age. Futures.

[CR20] Gamfeldt L, Snäll T, Bagchi R, Jonsson M, Gustafsson L, Kjellander P, Ruiz-Jaen MC, Fröberg M (2013). Higher levels of multiple ecosystem services are found in forests with more tree species. Nature Communications.

[CR21] Gregory R, Ohlson D, Arvai J (2006). Deconstructing adaptive management: Criteria for applications to environmental management. Ecological Applications.

[CR22] Gunderson LH, Holling CS, Light SS, Gunderson LH, Holling CS, Light SS (1995). Barriers broken and bridges built: A synthesis. Barriers and bridges to the renewal of ecosystems and institutions.

[CR23] Gunderson L (1999). Resilience, flexibility and adaptive management—Antidotes for spurious certitude?. Conservation Ecology.

[CR24] Gustafsson L, Baker SC, Bauhus J, Beese WJ, Brodie A, Kouki J, Lindenmayer DB, Lohmus A (2012). Retention forestry to maintain multifunctional forests: A world perspective. BioScience.

[CR25] Holling CS (1978). Adaptive environmental assessment and management.

[CR26] Kjellin, P. 2001. *Forest policy today*—*A description of the policies and other factors affecting forests and forestry.* Swedish Forest Agency Report 8B. Swedish Forest Agency, Jönköping (in Swedish).

[CR27] Larsson, S., T. Lundmark, and G. Ståhl. 2009. *Opportunities for intensive cultivation of forests.* Final report from the Government Commission 2008/1885. Uppsala: Sveriges lantbruksuniversitet. http://www.slu.se/Documents/externwebben/overgripande-slu-dokument/miljoanalys-dok/rapporter/Mint09/MINTSlutrapport.pdf (in Swedish).

[CR28] Larson, S., T. Lundmark, E. Mårald, and C. Sandström. 2014. *Preliminary study on the National Forestry Programme dialogue process*. Future Forests Report series 2014:2 http://www.slu.se/Global/externwebben/centrumbildningar-projekt/futureforests/Persons/FFRapport_NSP_2014-10-16.pdf (in Swedish).

[CR29] Leach M, Scoones I, Stirling A (2010). Dynamic sustainabilities; technology, environment, social justice.

[CR30] Lee KN (1999). Appraising adaptive management. Conservation Ecology.

[CR31] Lindenmayer DB, Likens GE (2010). The science and application of ecological monitoring. Biological Conservation.

[CR32] Lindenmayer DB, Likens GE (2010). Improving ecological monitoring. Trends in Ecology & Evolution.

[CR33] Lindenmayer DB, Hulvey KB, Hobbs RJ, Colyvan M, Felton A, Possingham H, Steffen W, Wilson K (2012). Avoiding bio-perversity from carbon sequestration solutions. Conservation Letters.

[CR34] Lindner M, Fitzgerald JB, Zimmermann NE, Reyer C, Delzon S, van der Maaten E, Schelhaas M-J, Lasch P (2014). Climate change and European forests: What do we know, what are the uncertainties, and what are the implications for forest management?. Journal of Environmental Management.

[CR35] Ludwig D, Mangel M, Haddad B (2001). Ecology, conservation, and public policy. Annual Review of Ecology and Systematics.

[CR36] McLain RJ, Lee RG (1996). Adaptive management: Promises and pitfalls. Environmental Management.

[CR37] Moir WH, Block WM (2001). Adaptive management on public lands in the United States: Commitment or rhetoric?. Environmental Management.

[CR38] Mårald E, Sandström C, Rist L, Rosvall O, Samuelsson L, Idenfors A (2015). Exploring the use of a dialogue process to tackle a complex and controversial issue in forest management. Scandinavian Journal of Forest Research.

[CR39] Norgaard RB, Kallis G, Kiparskya M (2009). Collectively engaging complex socio-ecological systems: Re-envisioning science, governance, and the California Delta. Environmental Science & Policy.

[CR40] Plummer R, Armitage D, Armitage D, Plummer R (2010). Integrating perspectives on adaptive capacity and environmental governance. Adaptive capacity and environmental governance.

[CR41] Plummer R (2013). Can adaptive comanagement help to address the challenges of climate change adaptation?. Ecology and Society.

[CR42] Puettmann KJ, Wilson SM, Baker SC, Donoso PJ, Drössler L, Amente G, Harvey BD, Knoke T (2015). Silvicultural alternatives to conventional even-aged forest management-what limits global adoption?. Forest Ecosystems.

[CR43] Ranius T, Caruso A, Jonsell M, Juutinen A, Thor G, Rudolphi J (2014). Dead wood creation to compensate for habitat loss from intensive forestry. Biological Conservation.

[CR44] Ranius T, Roberge JM (2011). Effects of intensified forestry on the landscape-scale extinction risk of dead wood dependent species. Biodiversity and Conservation.

[CR45] Reed MS (2008). Stakeholder participation for environmental management: A literature review. Biological Conservation.

[CR46] Reeves GH, Williams JE, Burnett KM, Gallo K (2006). The aquatic conservation strategy of the northwest forest plan. Conservation Biology.

[CR47] Rist L, Campbell BM, Frost P (2012). Adaptive management; where are we now?. Environmental Conservation.

[CR48] Rist L, Felton A, Samuelsson L, Sandström C, Rosvall O (2013). A new paradigm for adaptive management. Ecology and Society.

[CR49] Ruhl JB (2008). Adaptive management for natural resources—Inevitable, impossible, or both?. Rocky Mountain Mineral Law Institute.

[CR50] Ruhl, J.B., and R. Fischman. 2010. *Adaptive management in the courts*. FSU college of law, public law Research Paper No. 411; Indiana Legal Studies Research Paper No.154. http://ssrn.com/abstract¼1542632.

[CR51] Russell-Smith J, Whitehead PJ, Cook GD, Hoare JL (2003). Response of eucalyptus-dominated savanna to frequent fires: Lessons from Munmarlary, 1973–1996. Ecological Monographs.

[CR52] Sandström C, Lindkvist A, Öhman K, Nordström E-M (2011). Governing competing demands for forest resources in Sweden. Forests.

[CR53] Sandström C, Sténs A, Westholm E, Beland Lindahl K, Kraxner F (2015). Dilemmas in forest policy development—The Swedish forestry model under pressure. The future use of Nordic forests.

[CR55] Seidl R, Lexer MJ (2013). Forest management under climatic and social uncertainty: Trade-offs between reducing climate change impacts and fostering adaptive capacity. Journal of Environmental Management.

[CR56] SFA (2009). Rules on the use of exotic species.

[CR57] SFA. 2013a. *Forest Statistics Year Book**2013*. Swedish Forest Agency, Official Statistics of Sweden. Swedish Forest Agency, Jönköping, Sweden (in Swedish).

[CR58] SFA (2013). Adaptive forest management.

[CR59] SFA (2013). Preliminary study on the national forestry programme for Sweden—Proposals and positions.

[CR60] SFA. 2015. *Swedish forest act* http://www.skogsstyrelsen.se/Global/aga-och-bruka/Lagen/Skogsv%c3%a5rdslagstiftning2015webb.pdf.

[CR61] Shea K, Possingham HP, Murdoch WW, Roush R (2002). Active adaptive management in insect pest and weed control: Intervention with a plan for learning. Ecological Applications.

[CR63] SOU. 2007. *Sweden facing climate change: Threats and opportunities*. Swedish commission on climate and vulnerability. SOU 2007:60, http://www.regeringen.se/sb/d/574/a/96002.

[CR64] Stankey, G.H, R.N. Clark, and B.T. Bormann. 2005. *Adaptive management of natural resources: Theory, concepts, and management institutions.* General Technical Report PNW-GTR-654, US Department of Agriculture, Forest Service, Pacific Northwest Research Station, USA.

[CR65] Stankey CA, Henry G (2009). Adaptive environmental management: A practitioner’s guide.

[CR66] Stringer LC, Dougill AJ, Fraser E, Hubacek K, Prell C, Reed MS (2006). Unpacking “participation” in the adaptive management of social–ecological systems: A critical review. Ecology and Society.

[CR67] Temperli C, Bugmann H, Elkin C (2012). Adaptive management for competing forest goods and services under climate change. Ecological Applications.

[CR68] Ulmanen, J., Å.G. Swartling, and O. Wallgren 2012. *Climate change adaptation in Swedish forestry policy, a historical overview, 1990*–*2010.* Stockholm Environment Institute, Sweden. http://www.sei-international.org/mediamanager/documents/Publications/SEI-ProjectReport-Ulmanen-ClimateChangeAdaptation.pdf.

[CR69] Walters CJ (1986). Adaptive management of renewable resources.

[CR70] Walters CJ (1997). Challenges in adaptive management of riparian and coastal ecosystems. Conservation Ecology.

[CR71] Walters CJ (2007). Is adaptive management helping to solve fisheries problems?. Ambio.

[CR72] Walters CJ, Holling CS (1990). Large-scale management experiments and learning by doing. Ecology.

[CR73] Weiss G, Gadow K (2001). Administrating risk—A social science perspective on natural hazards prevention based on an Austrian case study. Risk analysis in forest management.

[CR74] Westley F, Holmgren M, Scheffer M (2010). From scientific speculation to effective adaptive management: A case study of the role of social marketing in promoting novel restoration strategies for degraded dry lands. Ecology and Society.

[CR75] Winkel G (2014). When the pendulum doesn’t find its center: Environmental narratives, strategies, and forest policy change in the US Pacific Northwest. Global Environmental Change.

[CR77] Young O (2009). Institutional dynamics: Resilience, vulnerability and adaptation in environmental and resource regimes. Global Environmental Change.

[CR78] Yousefpour R, Bredahl Jacobsen J, Jellesmark Thorsen B, Meilby H, Hanewinkel M, Oehler K (2011). A review of decision-making approaches to handle uncertainty and risk in adaptive forest management under climate change. Annals of Forest Science.

